# Large Dynamic Range Spectral Measurement in Terahertz Region Based on Frequency Up-Conversion Detection via OH1 Crystal

**DOI:** 10.3390/s24196245

**Published:** 2024-09-26

**Authors:** Jiasheng Yuan, Quanxin Guo, Xingyu Zhang, Naichang Liu, Xiaoqin Yin, Na Ming, Liyuan Guo, Binzhe Jiao, Kaiyu Wang, Shuzhen Fan

**Affiliations:** 1 Key Laboratory of Laser & Infrared System (Shandong University), Ministry of Education, Qingdao 266237, China; yuanjs1026@163.com (J.Y.); guo_qx@mail.sdu.edu.cn (Q.G.); xyz@sdu.edu.cn (X.Z.); yinxiaoqin@uor.edu.cn (X.Y.); 201920404@mail.sdu.edu.cn (N.M.); guoliyuan@mail.sdu.edu.cn (L.G.); jbzd163yx@163.com (B.J.); 2 Shandong Provincial Key Laboratory of Laser Technology and Application, School of Information Science and Engineering, Shandong University, Qingdao 266237, China; 202312632@mail.sdu.edu.cn (N.L.); 202212635@mail.sdu.edu.cn (K.W.); 3 School of Foundational Education, University of Health and Rehabilitation Sciences, Qingdao 266071, China; 4 Center for Optics Research and Engineering, Shandong University, Qingdao 266237, China

**Keywords:** terahertz spectral measurement, terahertz up-conversion detection, nonlinear frequency conversion, OH1 crystal

## Abstract

Terahertz spectroscopy systems, which integrate terahertz sources and detectors, have important applications in many fields such as materials science and security checking. Based on highly sensitive frequency up-conversion detection, large dynamic range spectral measurements in a terahertz region are reported. Our system realized the detection sensitivity at a 10 aJ level with a 2-(3-(4-hydroxystyryl)-5,5-dime-thylcyclohex-2-enylidene) malononitrile (OH1) crystal and a dynamic range up to seven orders. Based on this system, we verified the validity of the spectral measurement with tests which were conducted on monohydrate glucose, anhydrous glucose and mixed tablet samples with a thickness of 0.8 mm in 1~3 THz, respectively. Also, a mini coppery elbow tube with an inner diameter of 1 mm was used for the transmission of a terahertz wave to simulate some strip biological tissue samples. By allowing terahertz to transmit through this tube filled with 0.5 g glucose powder, we successfully obtained the absorption spectrum with a minimum transmittance at the absorption peak in the order of 10^−4^.

## 1. Introduction

A terahertz (THz) wave is an electromagnetic wave with a frequency between 0.1 THz and 10 THz. It is in the transition region between photonics and electronics and has many unique properties. A THz wave is biosafe and has good penetration of non-polar substances such as paper and cloth, and many molecules have “fingerprint” characteristics in the THz band [1,2,3,4,5]. This also provides a way to identify and measure some materials and biological tissues by THz spectroscopy. THz spectroscopy has developed rapidly in recent years, and has the characteristics of substance identification, being nondestructive, being non-contact and so on.

One of the most popular methods of THz spectral measurement is THz time-domain spectroscopy (THz–TDS). THz–TDS has been used in semiconductor identification [6], DNA [7], explosive detection [8] and so on. But a THz–TDS system usually needs an expensive femto-second laser source, and its effective detection dynamic range is usually about four orders of magnitude. Another commonly used THz spectroscopy measurement technique is Fourier transform far-infrared spectroscopy (FT-FIR). FT-FIR has been used in organic molecules and their conformations [9], explosive identification [10] and so on. But the dynamic range is generally not high enough for measurement, and the detector usually needs to work in a cryogenic environment. THz frequency up-conversion spectroscopy is another technology that has been studied in recent years, with the advantages of faster response and working at room temperature. By converting a THz wave into near-infrared (NIR) light through frequency up-conversion technology, more sensitive NIR detectors can be used to detect the up-converted signal. This can realize more sensitive detection and a larger dynamic range.

Many researchers have been studying the frequency up-conversion detection THz spectrum measurement. In 2014, H Minamide et al. used LiNbO_3_ crystal to obtain monochromatic THz radiation over a continuous tuning range of 0.7 to 2.9 THz and conducted relevant imaging measurements [11]. F Qi et al. used 4-dimethylamino-N-methyl-4-stilbazolium tosylate (DAST) crystal to achieve THz detection in the wide band of 1.85~30 THz using the nonlinear frequency up-conversion method. This work can realize wide frequency range THz spectrum measurement [12]. In 2022, Y Wang et al. used LiNbO_3_ crystal to obtain a 0.9~1.83 THz spectrum with frequency up-conversion technology [13]. In 2024, F Qi’s team achieved a continuous 4–32 THz emission spectrum of organic crystal DAST with a frequency resolution of 0.05 THz [14]. The above efforts had the ability of broadband THz spectral scanning, but did not conduct THz spectral measurements. In 2016, Kawase’s team realized a spectrum measurement system based on frequency up-conversion detection technology by using LiNbO_3_ crystal. The research group measured the spectrum of glucose, lactose and fructose in 1~2 THz, and obtained their respective characteristic spectra. They accurately identified sugars and mixed powders through packaging, demonstrating the ability of THz spectral measurement based on frequency up-conversion detection [15]. In 2023, K Murate in Kawase’s team used LiNbO_3_ crystal to generate a THz wave through an injection-seeded THz-wave parametric generator (Is–TPG) and conducted THz spectral measurements on maltose, glucose and lactose in a package that had high attenuation to THz waves [16].

In the current study, we integrated a THz source with nonlinear frequency conversion and an up-conversion detection system and constructed a 1~3 THz spectral measurement device by using OH1 crystal. Based on the detection sensitivity of 10 aJ and the detection technology of OH1 crystal THz frequency up-conversion with a dynamic range of seven orders of magnitude, we realized the THz spectrum measurement with a large dynamic range. To verify the validity of spectral measurement, we conducted THz spectral measurements on monohydrate glucose, anhydrous glucose and mixed tablet samples with a thickness of 0.8 mm in 1~3 THz, respectively, and we successfully obtained the THz absorption spectrum. It provided the basis for the subsequent sample measurements. We measured the THz absorption spectrum by wrapping the monohydrate glucose tablet in a sheet of A4 paper with a thickness of 0.1 mm to simulate the dimensions of letters, and successfully obtained the absorption spectrum and absorption peak. Common packaging materials, including paper, have high transmittance in the THz band, so through the THz spectrum system, samples can be easily detected and identified without damaging the packaging. We also used a mini coppery elbow tube with an inner diameter of about 1 mm and an interior coated with gold to simulate the measurement of some strip biological tissue. By allowing THz to pass through this tube filled with 0.5 g monohydrate glucose powder (in case of high loss), we successfully obtained the THz transmission spectrum with a minimum transmittance at the absorption peak in the order of 10^−4^.

## 2. Materials and Methods

**Figure 1:**
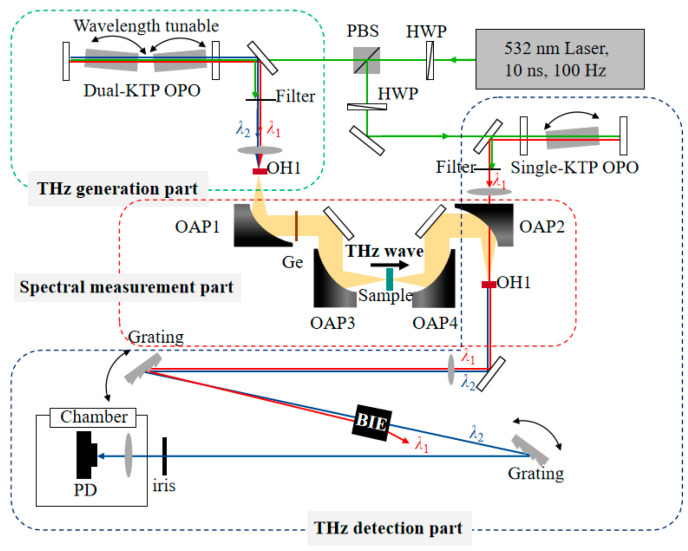
THz up-conversion spectrum measurement system based on OH1 crystal. PBS: polarizing beam splitter; HWP: half-wave plate; OAPs: off-axis parabolic mirrors; BIE: beam isolation enhancer; PD: photodetector; iris: aperture; The red arrows represent the optical paths of NIR light λ_1_; The blue arrows represent the optical paths of the NIR light λ_2_; The yellow area represents the THz transmission path; The green arrows represent the optical paths of the pump source with wavelength of 532 nm.

Figure 1 shows a schematic diagram of the experimental device. The system includes a THz generation part, a spectrum measurement part and a THz detection part. The laser source used in the experiment has a wavelength of 532 nm, a pulse width of 10 ns and a repetition rate of 100 Hz. The green laser is divided into two beams by a half-wave plate (HWP) and a polarizing beam splitter (PBS). The intensity of the two beams can be adjusted at the same time. One beam of the green laser incident is the THz generation part; the other beam of the green laser incident is the THz detection part.

The THz generation part is composed of a dual-KTiOPO_4_ (KTP) crystal optical parametric oscillator (OPO) [17] and an OH1 crystal. Two KTP crystals are fixed to the rotating motor which can control the rotation of the crystals by adjusting the rotation angle to control the optical wavelength of the OPO output. The wavelengths of two NIR beams λ_1_ and λ_2_ can be adjusted from 1250 to 1650 nm. THz waves can be generated through the difference frequency generation (DFG). The THz frequency ω_T_ satisfies the equation ω_T_ = ω_1_ − ω_2_, where ω_1_ = c/λ_1_ and ω_2_ = c/λ_2_ are the frequencies of the two NIR lights, respectively. The THz frequency tuning range is 1~3 THz in the OH1 crystal. Moreover, the THz frequency step of the spectral measurement is set as 0.05 THz. The frequencies of the two NIR lights are determined based on the phase matching of the OH1 crystal corresponding to a certain THz frequency.

The spectral measurement part is mainly composed of several off-axis parabolic mirrors (OAPs) and a germanium (Ge) plate with a thickness of 100 μm. The THz wave passes through OAP1 and transmits parallel to the Ge plate. The Ge plate is used to block the NIR lights λ_1_ and λ_2_ and has a transmittance of about 50% for 1~3 THz. Then, the THz wave passes through the spectral sample between OAP3 and OAP4 to complete the THz spectrum measurement of the sample.

The THz detection part is mainly composed of a pump laser λ_1_, an OH1 crystal and a detection light path. The pump laser λ_1_ is generated by a single KTP-OPO. The THz is focused on the OH1 crystal through OAP2 after the spectral measurement part. Meanwhile, the pump laser λ_1_ is also converged on the OH1 crystal through a focusing lens. According to the DFG, λ_1_ and the THz wave produce an up-converted signal λ_2_ in the OH1 crystal. λ_2_ is subsequently detected by the detector after transmitting the beam splitting device.

Because up-conversion detection is so sensitive, filtering out the non-signal light components in the experiment is also a priority. NIR lights λ_1_ and λ_2_ are collinear in space, so we used two grating devices to separate λ_1_ and λ_2_. As the THz frequency used is relatively low, the NIR wavelengths of λ_1_ and λ_2_ are relatively close. This results in a small separation angle of the dual grating devices. So, a beam isolation enhancer (BIE) component [18] is used to increase the separation angle of the NIR lights λ_2_ and λ_1_ and increase the signal-to-noise ratio (SNR).

To measure the minimum detectable THz energy and dynamic range of the system, we use a PIN detector (Thorlabs, Newton, NJ, USA, PDA10CF-EC), home-made avalanche photodiode (APD1, Bandwidth ~500 MHz, Peak response rate 6 × 10^5^ V/W) and home-made APD2 (Bandwidth ~700 MHz, Peak response rate 8 × 10^6^ V/W) as the detectors in our system. When the THz energy was high, we used the PIN detector and NIR attenuator to detect the up-converted signal. In contrast, when the terahertz energy was weak, we used the home-made APD to improve the sensitivity. Meanwhile, in order to compare with traditional THz detection methods, we also use the Golay cell (Tydex, St. Peterburg, Russia, GC-1D, optical responsivity with 110 kV/W) to measure the THz energy of the system.

To verify the feasibility of the spectral measurements of our experimental device, we conducted measurements on monohydrate glucose, anhydrate glucose and mixed sample tablets with a thickness of 0.8 mm in 1~3 THz, respectively. Various NIR attenuators were used when the up-converted signal was too high for the NIR detectors to make sure that the NIR detector worked in its operation range. The following signal was recorded as an equivalent signal voltage calculated as the readout signal multiplied by the attenuation ratio. To measure the noise level of the system, a 1 cm thick glass plate was used behind the Ge plate to block the THz wave. The noise voltage was given as the NIR detector’s output V_0_. The initial signal was recorded as the equivalent signal voltage V_1_. Then, we recorded the signal V_2_ by placing the sample between OAP3 and OAP4. The transmittance of the sample in the THz band could be obtained by the signal ratio (V_2_ − V_0_)/(V_1_ − V_0_). As the up-converted signal was strong enough, we used the PIN detector for all our subsequent measurements. The THz absorption spectrum of the sample in the 1–3 THz band was obtained through automated measurement by the program.

To simulate the measurement of items in external packaging, such as letters, a monohydrate glucose tablet wrapped in a 0.1 mm sheet of A4 paper was used. The characteristic absorption peak of monohydrate glucose was obtained successfully. In order to obtain a more accurate THz spectrum of the monohydrate glucose in A4 paper, we scanned the A4 paper and obtained the THz transmittance. By removing the influence of the A4 paper, we obtained the real THz spectrum of the monohydrate glucose.

**Figure 2:**
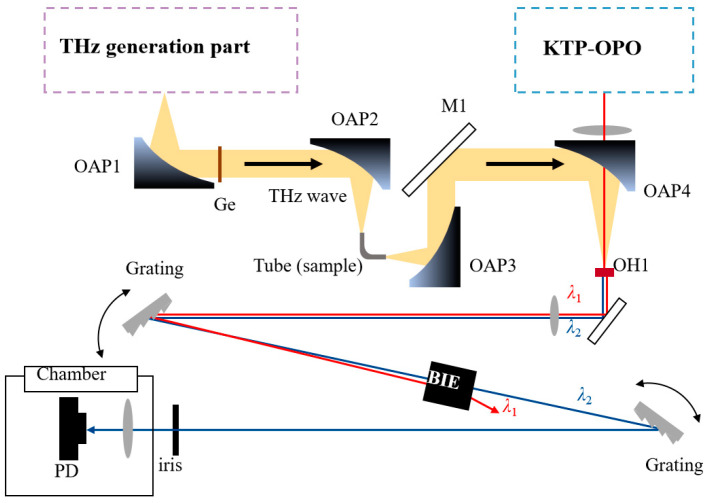
THz spectrum measuring device with a mini coppery elbow tube; M1: mirror; The red arrow represents the optical path of NIR light λ_1_; The blue arrow represents the optical path of the NIR light λ_2_; The yellow area (black arrow) represents the THz transmission path.

To simulate the measurement of some strip biological tissue, a mini coppery elbow tube was used for THz spectrum tests. This tube had an inner diameter of 1 mm and the interior was coated with gold to improve the THz transmission. The experimental design is shown in Figure 2. We put 0.5 g monohydrate glucose powder as the sample inside the tube. The THz wave was focused on the monohydrate glucose powder in the tube through dual OAP. Then, the NIR λ_1_ interacted with THz in the OH1 crystal to generate an up-converted signal which was received by the detector.

## 3. Results

### 3.1. THz Up-Conversion Sensitivity

**Figure 3:**
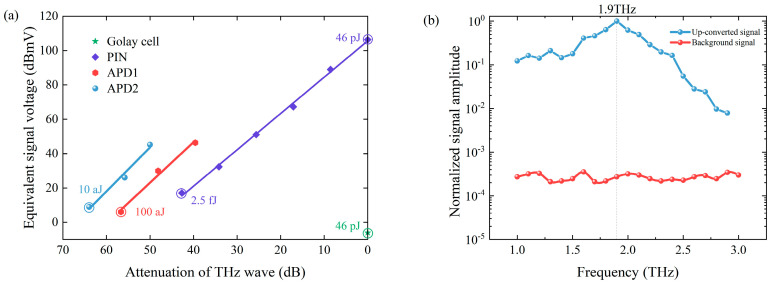
(**a**) Amplitude of different detectors as THz energy attenuates at 1.9 THz; (**b**) OH1 crystal output curve and background in 1~3 THz detected by PIN detector.

Using frequency up-conversion detection, THz waves can be converted into NIR light. Different NIR detectors have different sensitivities, which relate to the THz sensitivity. A PIN detector, home-made APD1, and APD2 were used as the detectors in our system to cover a wider range of THz energy measurements. Figure 3a shows the experimental results of these three detectors and a Golay cell at 1.9 THz. The THz energy without attenuation was 46 pJ, measured by the Golay cell. The value of 46 pJ was calculated by the optical responsivity of the Golay cell (corresponding to −6.02 dBmV). For up-conversion detection, lower THz energy was obtained by quantitative attenuation of THz energy. The attenuation ratio of THz energy is given as the horizontal axis in Figure 3a. NIR attenuators were also used for NIR detectors when the intensity of the up-converted signal was strong. The vertical axis in Figure 3a shows the equivalent amplitude calculated by dividing the readout signal of each detector by the NIR attenuation. When the signal was about the same as the background, the low detection limit of the detector was determined. In the measurement, the low detection limit was that THz energy attenuated by 63.98 dB when APD2 was used. Meanwhile, we obtained our minimum detection of the THz energy with a level of 10 aJ by dividing the THz energy by the attenuation ratio. The accuracy of these measurements was mainly related to the uncertainty of the terahertz attenuators, the noise of the Golay cell and the jitter of THz source. The THz attenuators have an uncertainty of about 2%. During the experiment, the output error of the Golay cell was about ±0.1 mV. For the jitter of the THz source, we minimized its impact on the measurement by taking multiple averages. By rationally using the NIR attenuator and changing the detector, we achieved large dynamic range measurements of seven orders of magnitude. Note that in all subsequent THz spectral measurements, only the PIN detector was used as its dynamic range was already sufficient for these tests.

Figure 3b gives the normalized frequency-upconverted signal from our system detected by the PIN detector using OH1 crystal. It shows the spectral measurement ability of 1~3 THz. The background measured after blocking the signal emitted from generator is also given in Figure 3b. It is composed of noise signal and residual pump light. Note that the signal shown in Figure 3b has already subtracted the background signal.

### 3.2. Validation of THz Spectral Measurement

**Figure 4:**
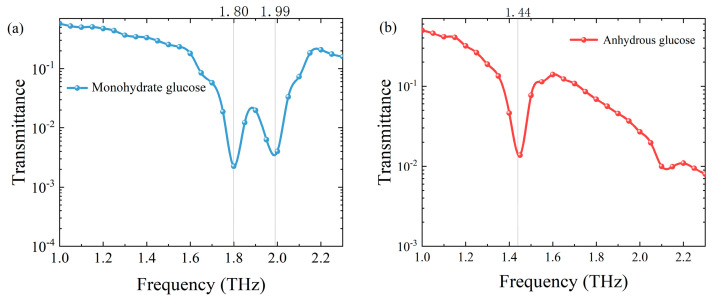
Absorption spectrum of (**a**) monohydrate glucose and (**b**) anhydrous glucose at THz band.

**Figure 5:**
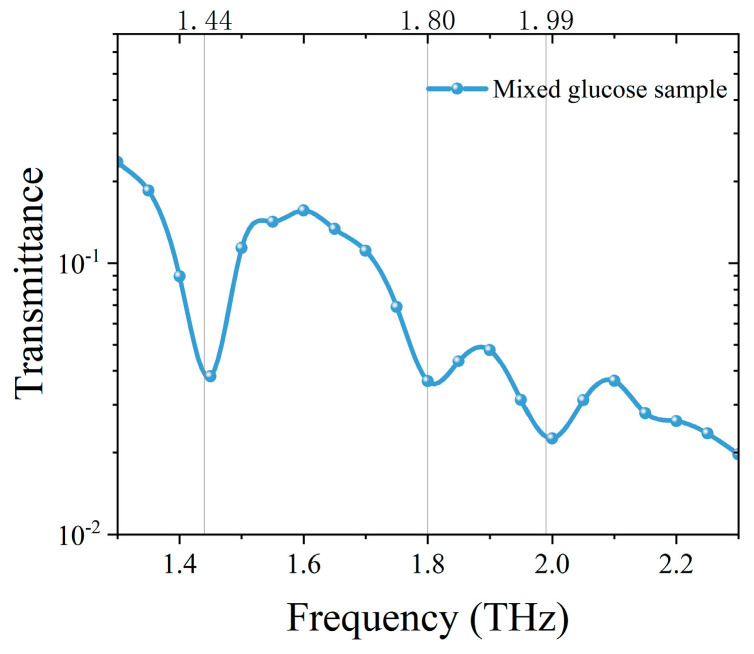
Absorption spectrum of mixed sample of monohydrate glucose and anhydrous glucose at THz band.

We measured the transmittance of monohydrate glucose and anhydrous glucose samples in the THz range to confirm their characteristic peak. The THz spectrum is shown in Figure 4. We could determine from the transmittance curve that the transmittance valley existed at the frequencies of 1.80 and 1.99 THz in monohydrate glucose and 1.44 THz in anhydrous glucose, which could be used as the characteristic peak for detection and identification. In order to further verify the detection and identification ability of the spectral system based on frequency up-conversion detection, we measured the mixed sample of monohydrate glucose and anhydrous glucose in the range of 1 to 2.3 THz. The transmittance is shown in Figure 5. The characteristic absorption peaks can be detected at 1.44 THz, 1.80 THz and 1.99 THz in the mixed sample, which indicate that the sample contains monohydrate glucose and anhydrous glucose components.

### 3.3. THz Detection of the Sample Wrapped in Paper

**Figure 6:**
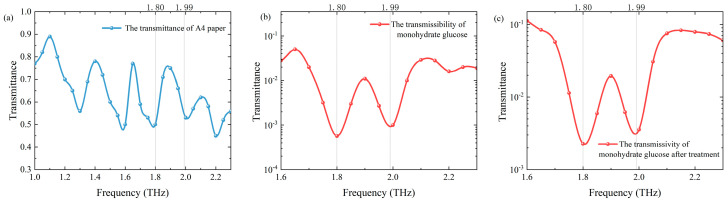
(**a**) The transmittance of A4 paper in the THz band. (**b**) THz spectrum of monohydrate glucose wrapped in A4 paper. (**c**) Calculating the THz spectrum of monohydrate glucose with the A4 paper packaging removed.

We carried out THz spectral measurement on the monohydrate glucose tablet packaged in A4 paper. Before the measurement, we conducted a THz spectral scan of A4 paper as the background as shown in Figure 6a. The THz spectrum of monohydrate glucose wrapped in A4 paper is shown in Figure 6b. Two absorption peaks of 1.80 THz and 1.99 THz are successfully identified. The transmittance is 5.7 × 10^−4^ and 1.0 × 10^−3^, respectively. In order to obtain the transmittance information of the packaged sample itself more accurately, we calculated and processed the transmittance of A4 paper and the overall sample as shown in Figure 6c. The result is basically consistent with the direct measurement of the monohydrate glucose sample.

### 3.4. THz Detection of the Sample in Waveguide

**Figure 7:**
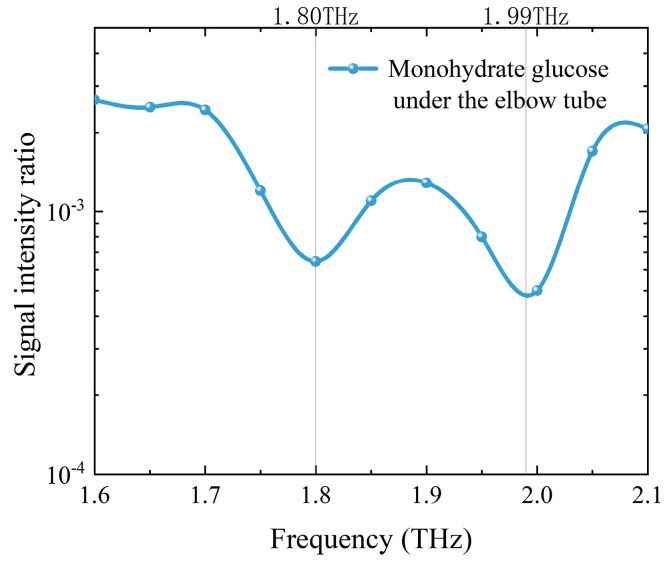
THz absorption spectrum of monohydrate glucose powder in the mini coppery elbow tube.

We placed 0.5 g monohydrate glucose powder inside the mini coppery elbow tube and placed the measuring device in the system optical path. We measured its frequency up-converted signal in the frequency range of 1.6 to 2.1 THz and compared it with the detection signal without the hollow waveguide. The THz absorption spectrum is shown in Figure 7. After THz passed through the tube, the amplitude of the THz up-converted signal was not high. Compared with the signal intensity of the detection system before and after placing the waveguide, the THz transmission efficiency was only about 5%. However, thanks to the high sensitivity of the frequency up-conversion detection system, the characteristic peaks of monohydrate glucose at 1.80 and 1.99 THz could still be obtained from the transmission spectrum. The transmittance was 6.5 × 10^−4^ and 5.0 × 10^−3^, respectively.

## 4. Discussion

In validation of THz spectral measurement, we measured anhydrous glucose with an absorption peak of 1.44 THz and monohydrate glucose with two absorption peaks of 1.80 and 1.99 THz. The result is basically the same as in previous work [19,20]. Because the frequency of THz waves is determined by the calculation of two beams of NIR light difference frequency, the accuracy of wavelength measurement of this system is relatively high. The error of THz frequency is mainly related to the resolution of the spectrometer and the spectral width of the pump light. The minimum spectral resolution of the spectrometer can reach 0.02 nm. The average spectral width of the pump wavelength is less than 0.2 nm. The error of the calculated THz frequency is about ±0.015 THz. The spectral measurement has a resolution of 0.05 THz. These values provide important information about the accuracy of the THz frequency calculation and the precision of the resolution in spectral measurement. It shows that our THz spectrum measurement results are accurate enough.

We simulated the THz spectral measurement of packaged monohydrate glucose with A4 paper and obtained the characteristic absorption peak of 1.80 THz and 1.99 THz. This implies that we can detect and identify the sample through its package. The transmittance at the two peaks of absorption in monohydrate glucose is 5.7 × 10^−4^ and 1.0 × 10^−3^. It is a challenge for many THz–TDS systems to measure such low THz energy. For our system, we can easily measure the signal by using the PIN detector. Moreover, our system has the potential to measure samples in thicker packaging, which means dealing with even weaker signals. By using several detectors, we are capable of conducting large dynamic range measurements of seven orders of magnitude.

In the detection of the sample in the waveguide, we successfully obtained the THz spectrum of the samples. This indicates that our waveguide measurement method is feasible. For specific biological samples, our system is adaptable and also capable of conducting measurements. Moreover, our high sensitivity and large dynamic range are well-suited for such measurements. Various specific THz sample boxes can be designed to meet the requirements of different applications.

Linearity is very significant for spectral measurement. In our system, the linearity between the THz energy and the up-converted signal intensity, as well as that between the optical signal input of the detector and the electrical signal output, remains to be determined. But, the calibrations of these two parts are relatively easy. In the future, we can determine the linearity of the detector by using various NIR attenuators to attenuate the up-converted signal and then determine the linearity between the detector and the up-converted signal by attenuating the THz energy. After calibration, the system can be expected to achieve good linearity and determine the intensity of THz based on the output amplitude of the detector and the transmittance of the NIR attenuator.

## 5. Conclusions

In summary, we designed a THz spectrum measurement device based on frequency up-conversion detection. Using the organic crystal OH1, THz pulse energy detection at the level of 10 aJ could be achieved at 1.9 THz and the dynamic range could reach seven orders of magnitude. We verified the feasibility of spectral measurement and successfully obtained the absorption peaks of monohydrate glucose and anhydrous glucose. The THz spectrum of monohydrate glucose was obtained successfully using A4 paper and a mini coppery elbow tube. The results show that our detection capability is sufficient and can be used for lower energy THz spectral detection. The feasibility of nondestructive testing and THz waveguide spectral measurement is also verified.

## Data Availability

The data underlying the results presented in this paper are not publicly available at this time but may be obtained from the corresponding author upon reasonable request.
